# Design of experiments as a tool to guide the preparation of tailor-made activated carbons

**DOI:** 10.1038/s41598-023-30642-8

**Published:** 2023-03-09

**Authors:** Jana B. Schaubeder, Chamseddine Guizani, Julian Selinger, Andreas Mautner, Michael Hummel, Stefan Spirk

**Affiliations:** 1 Institute of Bioproducts and Paper Technology (BPTI), Graz University of Technology, Inffeldgasse 23, 8010 Graz, Austria; 2 Department of Bioproducts and Biosystems, Aalto University, Vuorimiehentie 1, 02150 Espoo, Finland; 3 Institute for Materials Chemistry and Research, University of Vienna, Währinger Straße 42, 1090 Vienna, Austria; 4 VTT Technical Research Centre of Finland, VTT Finland, 1000, 02044 Espoo, Finland

**Keywords:** Chemical engineering, Energy science and technology, Engineering

## Abstract

Activated carbon produced from biomass exhibits a high specific surface area due to the natural hierarchical porous structure of the precursor material. To reduce production costs of activated carbon, bio-waste materials receive more and more attention, which has led to a steep increase in the number of publications over the past decade. However, the characteristics of activated carbon are highly dependent on the properties of the precursor material used, making it difficult to draw assumptions about activation conditions for new precursor materials based on published work. Here, we introduce a Design of Experiment methodology with a Central Composite Design to better predict the properties of activated carbons from biomass. As a model precursor, we employ well-defined regenerated cellulose-based fibers which contain 25 wt.% chitosan as intrinsic dehydration catalyst and nitrogen donor. The use of the DoE methodology opens up the possibility to better identify the crucial dependencies between activation temperature and impregnation ratio on the yield, surface morphology, porosity and chemical composition of the activated carbon, independent of the used biomass. The use of DoE yields contour plots, which allows for more facile analysis on correlations between activation conditions and activated carbon properties, thus enabling its tailor-made manufacturing.

## Introduction

As the most abundant natural polymer, cellulose has been studied extensively as renewable source for carbon materials. However, cellulose features some intrinsic properties that pose certain challenges when aiming for carbon materials. Cellulose is a linear polymer derived from D-glucose condensed through β(1 → 4)-glycosidic bonds. The repeating unit—anhydroglucose—denotes C_6_H_10_O_5_ resulting in a carbon content of 44.4 wt.%. Upon pyrolysis, cellulose undergoes a complex set of reactions occurring in parallel or sequence^1^. The fundamentals of cellulose pyrolysis have already been elucidated in the early 1960s by Tang and Bacon^2^. During this process, various volatile carbonaceous compounds can evolve reducing the carbon yield of the carbonized residue to 25 wt.% or even lower. One pathway to avoid substantial carbon loss is to promote the dehydration reaction, which can be catalyzed by substances also used as flame retardant agents^3,4^. Recently, it was shown that ionic liquid residuals also improve the carbon yield upon pyrolysis^5^. Although this could be attractive when aiming for precursor filaments spun from IL-solution, a thorough analysis of the costs of sacrificed IL is needed before this can be considered as a true asset of the process.

The catalytic activity of activated carbon is tightly connected to pore size distribution and specific surface area. To achieve these characteristics, the pre-carbonized biomass is typically subjected to an activation step. Both physical and chemical activation are common. For details, the reader is referred to the extensive review recently published by Thomas and coworkers ^6^. In short, physical activation is carried out using a reactant gas at temperatures ranging from 600 to 1200 °C, while chemical activation is carried out by mixing carbonaceous materials with chemical activating agents followed by heat treatment at 400–900 °C. The main advantages of chemical activation are higher yields, higher specific surface areas and lower activation temperatures. However, the disadvantages such as the corrosiveness of the activating agent and the necessity of an additional washing step after activation should be noted^7,8^. The most common agents for chemical activation include KOH^9,10^, ZnCl_2_^11–13^ or phosphoric acid compounds^14,15^.

The chemical activation agent KOH is most commonly used, as specific surface areas of over 2000 m^2^ g^−1^ can be achieved^16,17^. Although chemical activation with KOH is well-known, the activation mechanism has not been fully elucidated because of its complexity^8^. Otowa et al.^18^ suggested that there are four dominant reactions leading to the main products H_2_, H_2_O, CO, CO_2_, potassium oxide (K_2_O), and potassium carbonate (K_2_CO_3_), which have been widely accepted. The formation of these reaction products depends on activation parameters such as activation temperature and the precursor to KOH impregnation ratio, as well as on the carbon source itself and its reactivity, which in turn affect the resulting pore structure of the activated carbon. Consequently, it is difficult to trace the evolving structure^19^. In order to obtain the desired product, a systematic variation of the parameters for the activation protocol is required, but very rarely described in literature. Sophisticated data analysis tools such as Design of Experiment (DoE) are used in other contexts frequently and can provide global insights over the entire experimental range, also taking into account the interactions between parameters^20^.

In addition, the impact of the initial hierarchical morphology and potential impurities of the precursor materials on the activated carbon needs to be minimized or even excluded. Ideally, a well-defined precursor material, such as pure cellulose fibers should be used. In recent years, the Ioncell® process has been developed as an alternative to the viscose and Lyocell process for the production of man-made cellulosic fibers^21^. The main advantage of this process is its environmental friendliness due to the upcycling of waste fibers and the use of non-toxic chemicals. The ionic liquid 1,5-diazabicyclo[4.3.0]non-5-enium acetate ([DBNH]OAc) is used as a direct solvent for cellulose, which can be recycled by distillation after the spinning process, creating a circular process ^21,22^. In addition, Ioncell® fibers exhibit high orientation of cellulose chains along the molecular axis^23^ and are therefore an ideal candidate as precursor material for the production of well-structured activated carbon. Another advantage of this process is the possibility to produce composite fibers^24^, which allows the addition of heteroatoms such as nitrogen, which can act as an intrinsic dehydration catalyst alongside the ionic liquid residues and increase carbon yield.

In this study, the main novelty is to apply a Design of Experiment approach for the activation of chitosan doped Ioncell® fibers. These regenerated cellulose fibers can be obtained in our facilities on a pilot scale with high reproducibility. The main aim of this study was not to optimize treatment conditions for this type of fibers but to identify the crucial underlying linear and non-linear dependencies between temperature and amount of activation agent^25^ on one hand and carbon yield, specific surface area, pore size volume, carbon and oxygen contents on the other hand. The advantage of the approach is that it can be applied for different types of biomass, facilitating the manufacturing of activated carbons with defined structural and morphological properties.

## Materials and methods

### Precursor material

Ioncell® fibers containing 25 wt.% chitosan with an average molecular weight of 30 kDa (degree of deacetylation ≥ 90%, Glentham Life Sciences, UK) were prepared via dry-jet wet spinning of a cellulose-chitosan solution in an ionic liquid^21^ according to the methodology described by Zahra et al. resulting in fibers with a diameter of 16 µm^26^.

### Preparation of activated carbon from fibers

Fibers were cut into pieces of approx. 10 mm length and pre-carbonized at 400 °C with a heating rate of 5 °C min^−1^ and a holding time of 1 h under a constant nitrogen flow with a Tube Furnace (NBD-O1200-50IC, NBD Tech, China). The pre-carbonized fibers were mixed with an aqueous 30 wt.% KOH solution (Merck KGaA, Germany) with the corresponding mass ratio of pre-carbonized sample to KOH (Table 1) and dried in a drying oven (VWR, USA) overnight at 105 °C. The activation of the samples was carried out in a specially designed metal chamber, which was placed in a box furnace (N60/HR, Nabertherm, Germany) at the corresponding temperature for a holding time of 2 h with a heating rate of 5 °C min^−1^ in a nitrogen atmosphere. To remove all residuals of the activating agent, the activated fibers were washed in 100 ml 1:4 diluted HCl (37%, VWR, USA) for 4 h, then filtered with a MF-Millipore 0.8 μm MCE Membrane Filter (Merck KGaA, Germany) and washed with deionized water until neutral pH. The samples were then dried in a drying oven at 105 °C for 24 h. The denotation of the samples refers to AC (activated carbon) and the activation temperature. The K at the end of the sample name indicates the impregnation ratio C:KOH. An overview of the performed experiments is given in Table 1.

**Table 1:** Overview of the performed experiments.

Sample	T_activ_ (°C)	C:KOH
01-AC-700-K3	700	1:3
02-AC-800-K3	800	1:3
03-AC-900-K3	900	1:3
04-AC-700-K4	700	1:4
05-AC-800-K4	800	1:4
06-AC-900-K4	900	1:4
07-AC-700-K5	700	1:5
08-AC-800-K5	800	1:5
09-AC-900-K5	900	1:5
10-AC-800-K4	800	1:4
11-AC-800-K4	800	1:4

### Design of experiment

The concept of Design of Experiment^27^ (DoE) was employed to obtain information on the significance of activation temperature and impregnation ratio C:KOH (ratio of precursor to various amounts of KOH, 1:x) on the morphology and composition of the activated carbons. A face-centered central composite design with three replicates in the center point was chosen. The individual experiments were performed in random order to avoid introducing undesirable systematic effects. All mathematical operations were calculated with MATLAB® vR2020a. The experimentally obtained data were fitted to a six-parameter polynomial model (Eq. 1), where the responses $\widehat{Y}$ were expressed as a function of the two factors activation temperature *x*_1_ and C:KOH ratio *x*_2_.

1 $$\hat{Y} = b_{0} + b_{T} \cdot x_{1} + b_{C:KOH} \cdot x_{2} + b_{{T^{2} }} \cdot x_{1}^{2} + b_{{C:KOH^{2} }} \cdot x_{2}^{2} + b_{T \cdot C:KOH} \cdot x_{1} \cdot x_{2}$$

The significance of the model coefficients was validated using multiple linear regression, where the determination coefficients R^2^ were calculated for each model coefficient by omitting the term from the overall model. A minimum value for R^2^ of 0.80 is required to mark a good fit^28^. In addition, a t-test was performed to assess the significance of the model coefficients, which were displayed as error bars in the bar charts. If the error bar exceeds the zero line, the model coefficient is considered insignificant and was discarded to avoid overfitting. All parameters were recalculated subsequently. An overview of the discarded model coefficients for each response and the corresponding determination coefficients is given in Table S1. Oxygen ingress during activation caused experiment 03 to fail. Due to a lack of precursor fibers, the experiment was repeated twice with a lower amount of fibers, both times resulting in complete degradation of fibers during activation. Therefore, experiment 03 was excluded from the matrix, as the model coefficients and the response surfaces would otherwise be distorted. The measured responses for that experiment were therefore not discussed.

### Textural characterization of activated carbon using nitrogen adsorption manometry

Specific surface area and pore dimension measurements were performed with an automated gas adsorption analyzer (TriStar II 3020, Micromeritics, USA). After degassing the samples under a constant nitrogen flow at 170 °C overnight (FlowPrep 060, Micromeritics, USA), nitrogen adsorption experiments were carried out at 77 K. A duplicate determination was performed for all samples. The Brunauer–Emmett–Teller (BET) model was used for calculation of the SSA.

### Nanostructure characterization of activated carbon

Single Raman spectra were recorded with an inVia Qontor Raman system (Renishaw, UK) with a Leica HC PL FLUOTAR 100x/0.90 objective and a 532 nm laser. Triple measurements were performed with an exposure time of 10 s, a laser intensity of 1% and 10 accumulations per measurement. The spectra were smoothed using a moving average filter with a span of 9, baseline corrected (polynomial least square fit first order in the range of 600–700 cm^−1^ to 2000–2200 cm^−1^) and then normalized with respect to the G band height, using MATLAB® vR2020a.

### Elemental composition of activated carbon

Analysis of carbon, hydrogen, nitrogen, sulfur and oxygen content was performed by elemental analysis (EA) with a Flash Smart EA CHNS/O with MV (Thermo Scientific, USA). Each sample was measured twice. BBOT (2,5-(bis(5-tert-butyl- 2-benzo-oxazol-2-yl) thiophene) was used as standard for the CHNS analysis and a cystine standard was used for the oxygen analysis.

### Surface elemental composition of activated carbon by x-ray photoelectron spectroscopy

The elemental surface composition of activated carbon samples was analyzed with x-ray photoelectron spectroscopy (XPS) Nexsa XPS system (Thermo-Scientific, Massachusetts, USA) using Al Kα radiation operating at 72 W (beam diameter 400 µm) and an integrated flood gun. “Standard Lens Mode”, CAE analyzer Mode, a dwell time of 10 ms, pass energy of 200 eV, and an energy step size of 1 eV were used for the survey spectra. The surface of the specimens was cleaned by sputtering for 60 s with Ar-clusters (1000 atoms, 6000 eV, 1 mm raster size) prior to analysis. High-resolution spectra of C, O, N, and K were recorded with 50 passes at a pass energy of 50 eV, a dwell time of 50 ms and an energy step size of 0.1 eV. Quantification and analysis were executed with the software Thermo Avantage (v5.9914, Build 06617) using Smart background.

### Morphology of activated carbon by scanning electron microscopy

SEM images were recorded with a Zeiss Sigma VP (Zeiss, Germany) at low accelerating voltages (0.7–3.0 kV) using a Schottky FEG Emitter and an InLens or SE detector. Prior to imaging the samples were sputtered with gold for 30 s (20 mA) with a Q150R Plus rotary pumped coater (Quorum Technologies, UK).

## Results and discussion

### Mass yield of activated carbon

**Figure 1:**
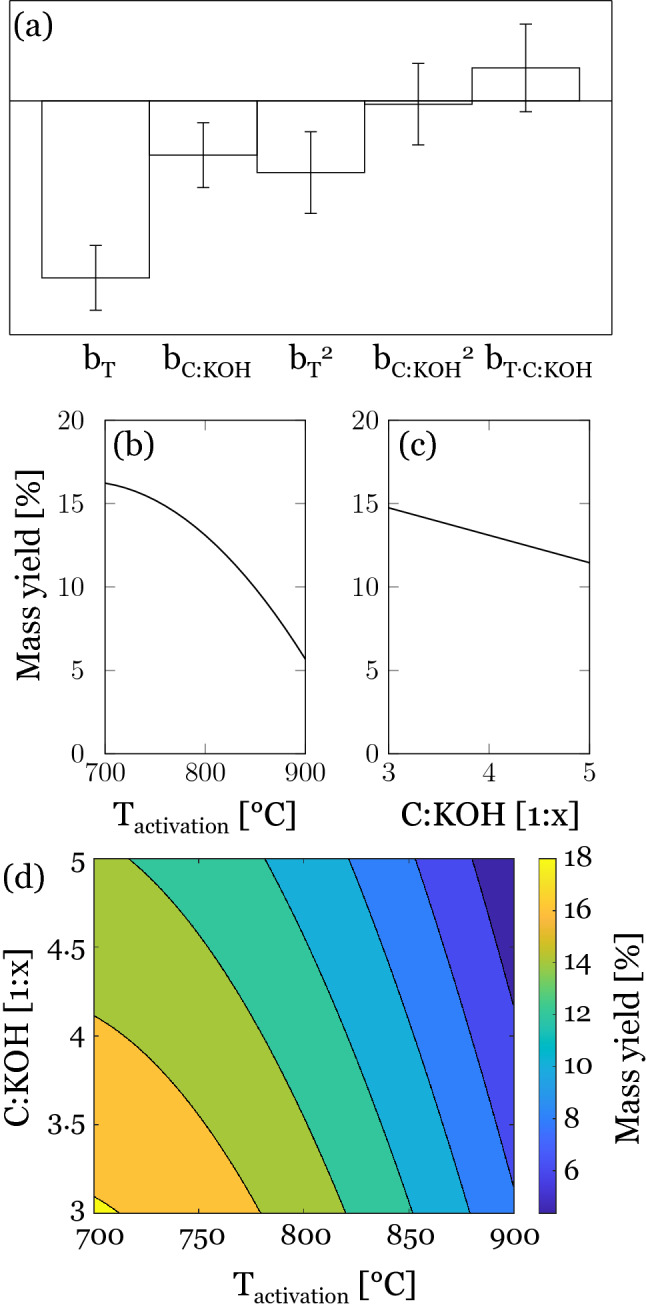
(**a**) Calculated model coefficients. Main effect plots of (**b**) activation temperature and (**c**) C:KOH ratio on the mass yield. (**d**) Response surface for mass yield (%).

The overall yield of the samples after the activation process ranged from 4.6 to 19.5% (Table 2). As expected, the mass yield decreased with increasing activation temperature. This is reflected by the relatively high, negative magnitude of the coefficients ${b}_{T}$ and ${b}_{{T}^{2}}$. Increasing the C:KOH ratio also causes a drop in the mass yield. However, the effect of C:KOH on the mass yield is less pronounced than for the temperature, which is reflected by the magnitude of the different model coefficients (Fig. 1a–c).

**Table 2:** Overall mass yield, specific surface area SSA_BET_, micropore area A_mic_, mesopore volume V_meso_, and elemental composition of the activated carbons determined by elemental analysis EA and XPS.

Sample	Yield	Specific surface area (m^2^ g^−1^)		Pore volume (cm^3^ g^−1^)	Elemental composition^b^					
	(%)^a^	SSA_BET_	A_mic_	V_meso_	C_EA_ (%)	N_EA_ (%)	O_EA_ (%)	C_XPS_ (at.%)	N_XPS_ (at.%)	O_XPS_ (at.%)
01-AC-700-K3	19.5	1792	1371	0.083	86.1	0.79	6.2	78.2	0.71	20.1
02-AC-800-K3	13.9	2184	1046	0.235	90.8	0.47	3.8	80.9	0.93	17.0
04-AC-700-K4	15.2	1968	1197	0.134	87.7	0.91	5.0	86.6	0.98	11.2
05-AC-800-K4	14.0	2826	552	0.662	91.9	0.39	4.3	84.8	0.8	13.2
06-AC-900-K4	6.1	2844	632	1.178	92.7	0.23	2.6	94.8	0.27	4.4
07-AC-700-K5	13.9	2245	907	0.354	88.4	0.61	5.5	90.8	0.56	7.6
08-AC-800-K5	11.4	2973	560	0.944	93.4	0.36	3.5	88.2	0.51	10.3
09-AC-900-K5	4.6	3019	666	1.608	93.6	0.22	2.1	96.9	0.23	2.9
10-AC-800-K4	11.6	2539	725	0.486	91.7	0.53	3.8	89.5	0.58	8.9
11-AC-800-K4	14.5	2517	754	0.402	92.9	0.32	3.3	89.6	0.46	8.9

^a^Calculated by dividing the final mass of washed and dried activated carbon by the initial mass of precursor fiber.

^b^C_EA_, N_EA_ and O_EA_ percentages were calculated excluding the hydrogen data for better comparability to XPS data.

While most of the volatile components of the precursor were already released during the pre-carbonization process, the gasification reactions of the carbon accelerate with increasing activation temperature, which drastically decreased the mass yield^29^. The decrease in mass yield with increasing C:KOH ratio may result from KOH induced catalytic oxidation reactions^30^. However, it is also stated in literature that the addition of KOH may delay carbon degradation, which could explain the less prominent effect of the C:KOH ratio on the mass yield^31^. The response surface shown in Fig. 1d indicates a combined dependence of activation temperature and C:KOH ratio on the mass yield at temperatures up to 850 °C, while above this the effect of the activation temperature outweighs the effect of C:KOH ratio. The highest yield of 19.5% was achieved under the mildest activation conditions (700 °C and C:KOH 1:3).

### Characterization of the activated carbon texture using nitrogen adsorption manometry

Nitrogen physisorption isotherms of all samples at 77 K are shown in Fig. S1. According to the IUPAC classification^32^, the isotherms of samples 01-AC-700-K3, 02-AC-800-K3 and 04-AC-700-K4 (low activation temperatures and low C:KOH ratios) are of type I, which are defined by microporous solids where the limited uptake is determined by the accessibility of the micropore volume instead of the internal surface area. Samples 05-AC-800-K4, 06-AC-900-K4, 07-AC-700-K5, 08-AC-800-K5 and 09-AC-900-K5 show type IV isotherms with type H4 hysteresis loops, which can be associated with capillary condensation in mesopores indicating a mesoporous nature of the solids. This is consistent with literature, which states that increased C:KOH ratios (> 1:4) evidently broaden the micropores^8^. In another study, carbon fabrics from viscose fibers were activated with a similar activation protocol and also show type IV isotherms for samples with C:KOH 1:5 and activation temperatures of 700 °C^33^. Furthermore, the shape of the hysteresis loop is often linked to specific pore structures and the type H4 loop is assumed to have narrow, slit-like pores. The most commonly used model to characterize porous carbon pore size distribution (PSD) is the infinite slit-shaped pore model. This model assumes graphite-like infinitely extended pore walls, although the impact of finite sizes and pore terminations has been recognized and novel, improved methods have already been developed^32,34^. The values experimentally obtained for the BET specific surface area SSA_BET_, micropore area A_mic_ according to the t-plot method and mesopore volume V_meso_ according to the Dollimore–Heal adsorption model are given in Table 2.

**Figure 2:**
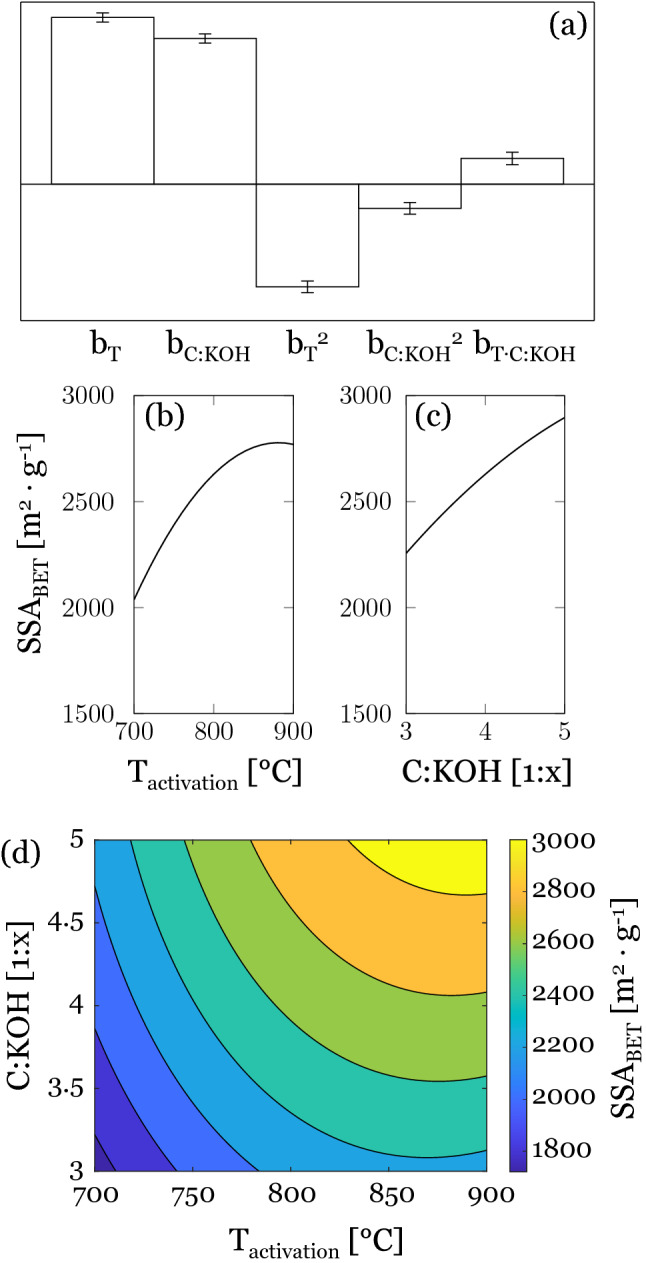
(**a**) Calculated model coefficients for SSA_BET_. Main effect plots of (**b**) activation temperature and (**c**) C:KOH ratio on the specific surface area. (**d**) Response surface for SSA_BET_ (m^2^ g^−1^).

The SSA_BET_ values obtained for our samples exceeded those of commercial activated carbons, which are typically in a range between 500 and 2000 m^2^ g^−1^^35^. The calculated model coefficients (Fig. 2a) show that activation temperature and C:KOH ratio both have a strong influence on the increase of SSA_BET_, whereas no significant interactions between the factors can be observed. The model suggests a maximum activation temperature close to 900 °C beyond which the SSA_BET_ decreases again (Fig. 2b). Throughout the experimental domain an increase in C:KOH ratio leads to a continuous increase in SSA_BET_ (Fig. 2c). Similar results have been reported in literature for the chemical activation of sewage sludge with H_2_SO_4_, where the SSA_BET_ was continuously increasing with increasing C:KOH ratio, while the activation temperature displayed a maximum at already 700 °C^36^. Muniandy et al.^37^ reported similar effects for activated carbons from rice husk. In that study, the highest specific surface area of 2696 m^2^ g^−1^ was achieved with a C:KOH ratio of 1:5 and an activation temperature of 850 °C. Exceeding either value decreases SSA_BET_, which was explained by higher gasification rates at higher temperatures leading to an enlargement of microporosity and thus lowering the SSA_BET_. In the study on the activation of viscose fibers mentioned above, an increase in SSA with increasing C:KOH ratio was likewise observed (T_activ_ 700 °C, C:KOH 1:4 and 1:5), but with notably lower SSAs than in this work^33^. In the response surface (Fig. 2d) maximum values of over 3000 m^2^ g^−1^ are located in the upper right corner, which means that highest specific surface areas can be reached under harshest activation conditions (T_activ_ 900 °C and C:KOH 1:5). It should be noted, however, that SSA values above 2000 m^2^ g^−1^ should be viewed critically, as they can be easily overestimated due to capillary condensation and pore filling phenomena.

**Figure 3:**
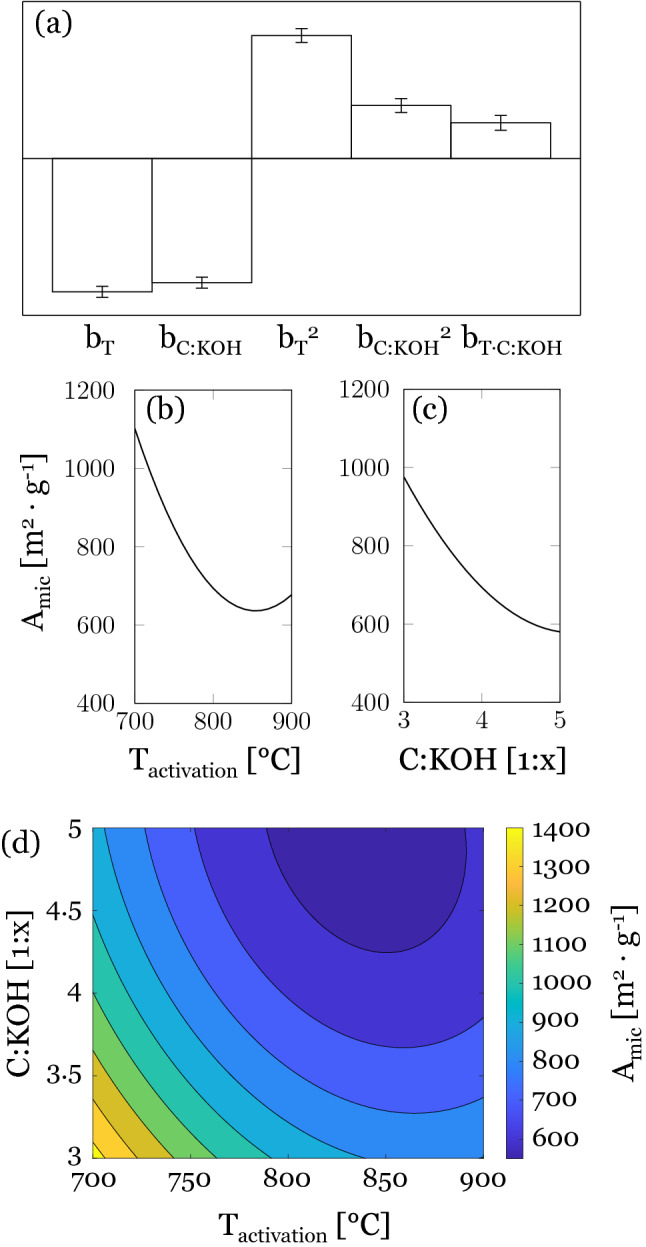
(**a**) Calculated model coefficients for A_mic_. Main effect plots of (**b**) activation temperature and (**c**) C:KOH ratio on the micropore surface area according to the t-plot method. (**d**) Response surface for A_mic_ (m^2^ g^−1^).

A t-plot analysis was used to determine the micropore surface areas A_mic_ of the activated carbons. However, this method is prone to underestimation of A_mic_, which is why the data should be regarded as comparative values rather than absolute values^32,38^. The obtained micropore surface areas ranged between 552 and 1371 m^2^ g^−1^. The calculated model coefficients show that A_mic_ is decreasing with increasing activation temperature and C:KOH ratio and that there is a non-negligible interaction between the factors (Fig. 3a). The main effect plot of activation temperature (Fig. 3b) shows a minimum at about 850 °C and it appears that with increased C:KOH ratio A_mic_ grows towards a minimum at the extreme right edge of the plot (Fig. 3c). Comparing the results with the micropore surface areas for the activated viscose fibers in the study mentioned above, the values for the AC activated at 700 °C with a C:KOH ratio of 1:4 are in the same range, while interestingly, an opposite effect was established for the sample with a higher C:KOH ratio of 1:5. The viscose fibers had a holding time of only 45 min during the activation process, which might prevent the micropores from expanding as much, resulting in higher micropore surface areas^33^. The response surface (Fig. 3d) shows a minimum in the upper right corner of the experimental region, which means that micropore surface areas are smallest under the harshest activation settings. This can be explained by pore broadening under such conditions, resulting in the formation of mesopores and consequently a decrease in micropore surface area. The highest micropore surface area of about 1400 m^2^ g^−1^ was achieved only for a narrow region under the mildest activation conditions (T_activ_ 700 °C and C:KOH 1:3).

**Figure 4:**
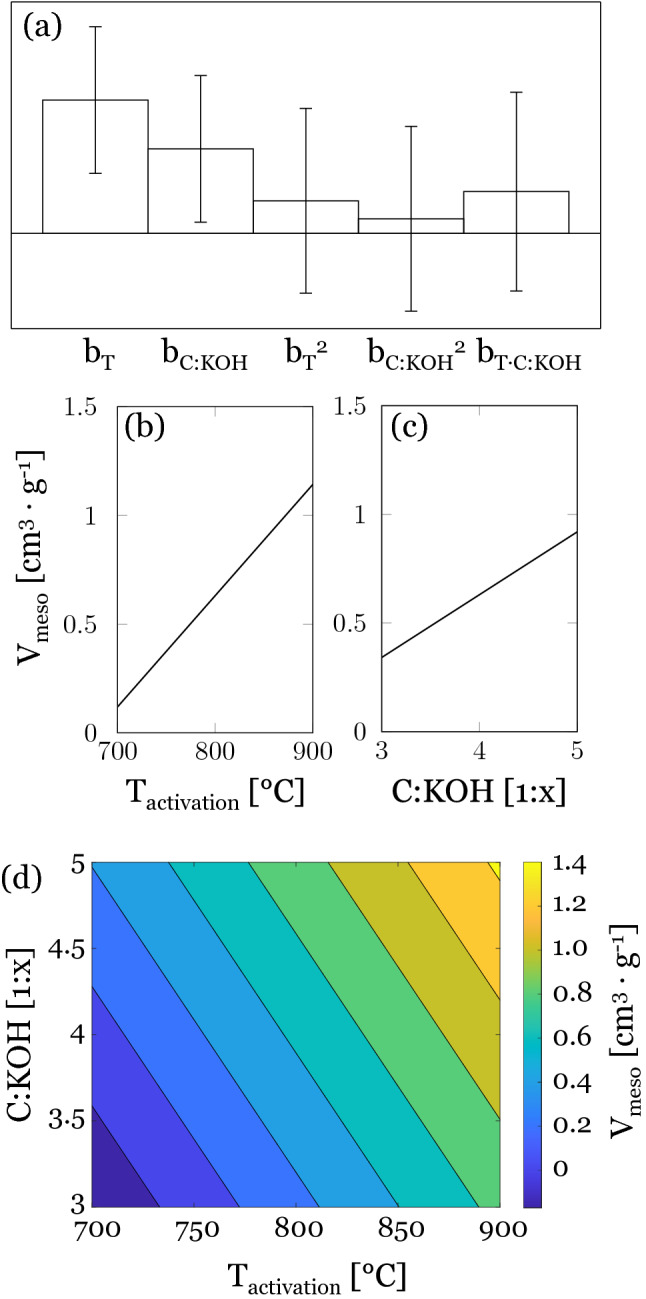
(**a**) Calculated model coefficients for V_meso_. Main effect plots of (**b**) activation temperature and (**c**) C:KOH ratio on the mesopore volume according to Dollimore-Heal adsorption data. (**d**) Response surface for mesopore volume (cm^3^ g^−1^).

The mesopore volume V_meso_ was calculated from both Dollimore-Heal (DH) and Barrett, Joyner and Halenda (BJH) adsorption data. As the data were in excellent agreement, only the mesopore volumes calculated from DH adsorption data are presented below. The obtained mesopore volumes ranged from 0.083 to 1.608 cm^3^ g^−1^. The model suggests a linear increase in mesopore volume with increasing activation temperature and C:KOH ratio, where activation temperature has a stronger effect than C:KOH ratio (Fig. 4a–c). Compared to viscose fibers reported in literature, the two comparable samples to 04-AC-700-K4 and 07-AC-700-K5 had mesopore volumes of 0.07 and 0.71 cm^3^ g^−1^, respectively^33^. For the former sample, approximately twice the mesopore volume (V_meso_: 0.134 cm^3^ g^−1^) was determined in this work, while for the latter sample only half the mesopore volume (V_meso_: 0.354 cm^3^ g^−1^) was obtained. However, both show an upward trend with increasing C:KOH ratio. In the response surface (Fig. 4d) a narrow region for a maximum mesopore volume of about 1.4 cm^3^ g^−1^ is located at the harshest activation conditions (T_activ_ 900 °C and C:KOH 1:5). From there, V_meso_ decreases linearly to the mildest conditions.

The PSD according to the Dollimore-Heal adsorption data (Fig. S2) shows that with increasing activation temperature and C:KOH ratio, the pores broadened, the pore volumes increased and peak maxima shifted to higher pore diameters, underlining the assumptions from above. Micropore surface area decreased with harsher activation conditions and showed a minimum at 850 °C and a C:KOH ratio of almost 1:5, presumably due to the enlargement of the micropores and the resulting reduced micropore surface area. Furthermore, the specific surface area increased with harsher activation conditions throughout the experimental region. The mesopore volume follows a linear positive trend towards harsher activation conditions. In a study about the influence of pre-carbonization the same trend is visible, suggesting that pore-drilling and pore-widening occur simultaneously up to a certain temperature and increase both micro- and mesopore volumes, whereas above that temperature pore-widening effect exceeds the pore drilling effect by destroying the pore walls of micropores^39^.

### Elemental composition of activated carbon

The carbon, nitrogen and oxygen contents of the activated carbons were determined by elemental analysis (EA) and X-ray photoelectron spectroscopy (XPS) (Table 2). Furthermore, the carbon (43.4%) and nitrogen (1.7%) fractions of the precursor fibers determined by EA were used to calculate the carbon to nitrogen ratio (C/N). For pure chitosan, the C/N value (5.15) was calculated according to the molecular formula (C_6_H_11_NO_4_)^40^. As precursor fibers used contain 25 wt.% chitosan, and cellulose and chitosan have the same number of carbon atoms, one quarter of the carbon mass determined by EA is attributable to the chitosan content. Hence, the C/N ratio of the untreated precursor fibers can be calculated according to Eq. 2, resulting in a value of 6.29, which means that the precursor fibers actually contain slightly less than 25% chitosan, most likely caused by chitosan losses during the spinning process.

2 $$\frac{C}{N} = \frac{1}{4} \cdot \frac{{m_{\% C} }}{{m_{\% N} }}$$

The obtained nitrogen contents determined by EA and XPS are in good agreement and range from 0.22 to 0.91% and 0.23 to 0.98 at.%, respectively. However, it should be noted that BBOT, the standard used for CHNS analysis, has a much higher nitrogen content than the activated carbons. Therefore, the data must be treated with caution. Applying the DoE model an unsatisfactory determination coefficient was obtained for the responses obtained from EA and is therefore not discussed. Furthermore, the responses obtained from XPS measurements could not be fitted to the model, since all model coefficients were assessed insignificant. However, the conducted measurements gave a rough estimation of the nitrogen content left after chemical activation. Liu et al.^41^ determined a nitrogen content of 2.10 and 0.43% with EA after chemical activation of pure chitosan at activation conditions of 850 °C/C:KOH 1:3 and 950 °C/C:KOH 1:2, respectively. Considering that the precursor fibers in this work consisted of only one quarter of chitosan, the values match fairly well. Furthermore, Lin et al.^42^ found that the nitrogen content in activated carbons prepared from a nitrogen-rich precursor material decreases considerably from 2.55 to 1.37 at.% upon chemical activation with KOH (T_activ_ 800 °C/C:KOH 1:3). This underlines the assumption that considerably higher initial nitrogen contents would have been required in the precursor fibers to maintain substantial nitrogen content after chemical activation.

**Figure 5:**
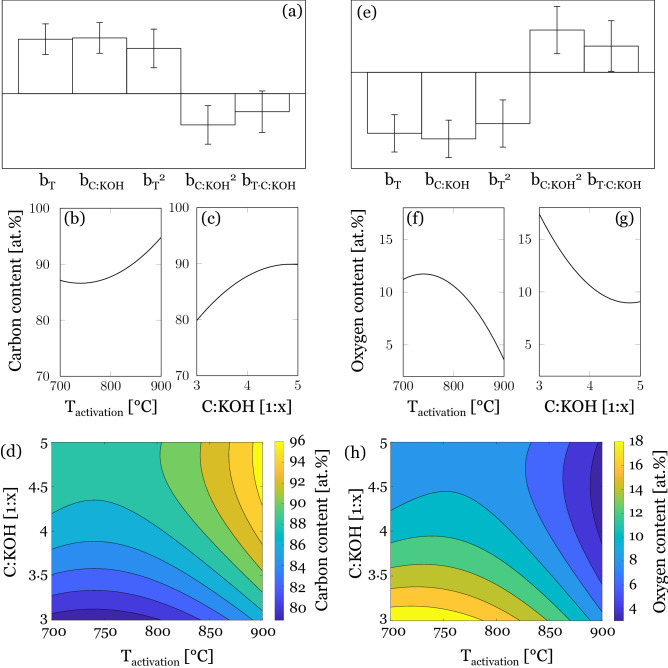
(**a**) Calculated model coefficients for carbon content determined by XPS. Main effect plots of (**b**) activation temperature and (**c**) C:KOH ratio on the carbon content of the activated carbons. (**d**) Response surface for carbon content determined by XPS (at.%). (**e**) Calculated model coefficients for oxygen content determined by XPS. Main effect plots of (**f**) activation temperature and (**g**) C:KOH ratio on the oxygen content of the activated carbons. (**h**) Response surface for oxygen content determined by XPS (at.%).

The carbon content of the activated carbon determined by EA ranges from 86.1 to 93.6%, indicating a high carbonization of the activated carbons (Fig. S3). The carbon content is slightly increasing with increasing C:KOH ratio, however, carbon content is more dominantly dependent on activation temperature. A maximum carbon content was found in the response surface around 860 °C at a C:KOH ratio of 1:5 (Fig. S3d). The surface carbon content determined by XPS range between 78.2 and 96.9 at.% in the experimental domain (Fig. 5). Surface carbon content is increasing with increasing activation and increasing C:KOH ratio, showing a minimum at low activation temperatures of around 750 °C and a maximum at high C:KOH ratios of 1:5 (Fig. 5d). For the aforementioned chemical activation of viscose fibers at a temperature of 700 °C, carbon contents of 84.5 and 88.5% are determined by EA and XPS, respectively, which are within the same range^33^. The response surface (Fig. 5d) shows that high carbon content above 88 at.% is reached at activation temperatures above 850 °C, while a sufficiently low C:KOH ratio of 1:3.5 is enough.

The oxygen content of the activated carbon determined by EA ranges between 2.1 and 6.2% and the model indicates a sole influence of activation temperature on oxygen content, thus a linear decrease from 700 to 900 °C (Fig. S4). The surface oxygen content determined by XPS ranges between 2.9 and 20.1 at.%. Therefore, higher oxygen contents were observed on the surface of the activated carbon. For the chemical activation of the aforementioned viscose fibers at a temperature of 700 °C, oxygen contents of 12.3 and 11.5% were determined by EA and XPS, respectively, which is in the range of the values obtained by XPS in this study^33^. Unlike for the oxygen content determined by EA, the model suggests that the surface oxygen content is decreasing with both, higher activation temperature and C:KOH ratio Fig. 5e–g). The response surface and the prediction plots (Fig. 5f–h) showed the opposite behavior than for the surface carbon content (Fig. 5b–d). Maximum surface oxygen content was obtained at the mildest activation conditions, while minimum surface oxygen content was observed at activation temperatures above 850 °C, which can be correlated with accelerated gasification reactions and thus increased CO loss, which also decreases the overall yield. The correlation between the oxygen content of activated carbon fibers and the yield was confirmed by a study on wood sawdust^43^.

### Morphology of activated carbon

**Figure 6:**
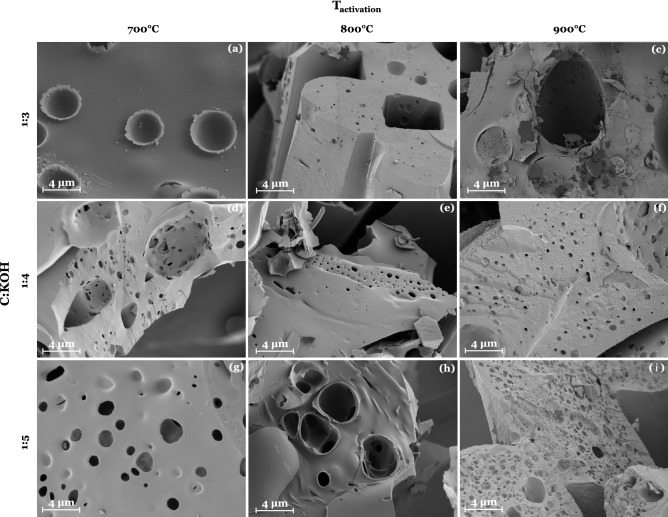
SEM images of all activated carbons according to the design matrix with a magnification of 5 K X. Samples: (**a**) 01-AC-700-K3, (**b**) 02-AC-800-K3, (**c**) 04-AC-700-K4, (**d**) 05-AC-800-K4, (**e**) 06-AC-900-K4, (**f**) 07-AC-700-K5, (**g**) 08-AC-800-K5, and (**h**) 09-AC-900-K5.

Fiber fragments are present in the rough surface morphology of all activated carbons, but they vary in size depending on activation temperature and C:KOH ratio (Fig. S5). If the activation temperature and the C:KOH ratio are increased, the activated carbons appear to become more brittle and exhibit significantly smaller fiber fragments. At higher magnification of 5 K X (Fig. 6) the difference in quantity and size of macropores can be observed. Sample 01-AC-700-K3 (Fig. 6a) has mainly a crater-like surface structure, while sample 09-AC-900-K5 (Fig. 6h) has a large number of seemingly small pores. Deep-reaching channels of only 1 µm in diameter appear to form solely in samples with a C:KOH ratio of 1:5.

Figure 6b shows fiber fragments that appear to be fused together. This effect has also been observed and reported in literature and originates from the removal of hydroxyl groups during pre-carbonization, leading to a reduction in intermolecular hydrogen bonding and thus the likelihood of fibers melting together during pyrolysis, which can be prevented by the use of flame retardant agents^44^.

### Nanostructure of activated carbon

One representative Raman spectrum per sample normalized to the G band and color-coded according to the activation temperature is shown in Fig. S6. In general, the spectra are dominated by two bands, one at a Raman shift of 1380 cm^−1^, called the D band (D for disorder) and one at 1610 cm^−1^, called the G band (G for graphitic)^45^. The D band is associated with defects and disorder of carbon materials, whereas the G band reflects the bond stretching of sp^2^ carbon atoms^42^. Thus, the degree of carbonization/graphitization can be represented by the intensity ratio of I_D_/I_G_. Graphitization usually only occurs at high temperatures (e.g., 3000 °C) and is basically a physical process connected with crystalline growth, whereas carbonization is mainly related to chemical processes where heteroatoms are eliminated and a pure sp^2^ carbon material is formed^46^. Since only temperatures of up to 900 °C are used in this work, the change in I_D_/I_G_ corresponds to carbonization reactions. In addition, a hidden band at about 1500 cm^−1^ is suggested in the literature to be attributed to the high signal intensity between the I_D_ and I_G_ band maxima and is referred to as the D” band, originating from amorphous domains, functional groups, interstitial defects and point-like sp^2^ based defects, especially in non-hexagonal rings^45,47,48^.

The calculated I_D_/I_G_ ratios (Table S2) were fitted to the DoE model, however, all model coefficients were assessed insignificant. When looking at the data, an increase in I_D_/I_G_ ratio can be observed with increasing activation temperature, which can be attributed to carbonization^46^. The Raman spectra of all samples look alike (Fig. S6), however, samples 09-AC-900-K5 and 02-AC-800-K3 show somewhat higher and narrower D bands and sample 06-AC-900-K4 shows only a narrower D band. The I_D_ maxima of the remaining samples display the same intensities. Differences can be observed for the presumed D” band at 1500 cm^−1^. Samples 06-AC-900-K4 and 02-AC-800-K3 show the lowest local minimum and thus the weakest D” band followed by samples 09-AC-900-K5 and 01-AC-700-K3. The remaining local minima show the same intensities. From the obtained spectra no clear trend was discernible, which could be due to opposing effects of activation temperature and C:KOH ratio on the nanostructure of the activated carbons.

## Conclusion

Central Composite Design in DoE was employed to better predict the properties of activated carbons from a well-defined cellulose source, regenerated cellulose fibers. The intention of this work was to showcase optimal conditions for the preparation of activated carbons from these particular cellulose fibers. We showcase that the DoE methodology is a suitable tool to better identify the underlying, partially non-linear, dependencies between the decisive activation parameters. The resulting contour plots show that depending on the intended use of the activated carbon, one should consider the parameters required for good performance of the product and adjust the activation conditions accordingly in order to obtain a higher quality product, taking into account the economics of production. These contour plots are invaluable as they provide a map, guiding to choose the experimental conditions to achieve the desired properties of the activated carbons.

## Supplementary Information

Supplementary Information.

## Data Availability

The used MATLAB® code and all the other experimental data will be made available upon request by S. Spirk.
