# Supplementary material for: Design of experiments as a tool to guide the preparation of tailor-made activated carbons
Source: Sci Rep. 2023 Mar 9;13:3977. doi: 10.1038/s41598-023-30642-8 (PMC9998870; doi:10.1038/s41598-023-30642-8)
Supplement: Supplementary file 1 — Supplementary Information. [file 41598_2023_30642_MOESM1_ESM.docx]

**Design of Experiments as a tool to guide the preparation of tailor-made activated carbons**

*Jana B. Schaubeder^a^, Chamseddine Guizani^b^, Julian Selinger^a,b^, Andreas Mautner^c^, Michael Hummel^b^*, Stefan Spirk^a*^*

*^a^Graz University of Technology, Institute of Bioproducts and Paper Technology (BPTI), Inffeldgasse 23,* *8010 Graz, Austria: jana.schaubeder@tugraz.at, julian.selinger@tugraz.at, stefan.spirk@tugraz.at*

*^b^ Department of Bioproducts and Biosystems, Aalto University, Vuorimiehentie 1,* *02150 Espoo, Finland: chamseddine.guizani@aalto.fi, michael.hummel@aalto.fi*

*^c^Institute for Materials Chemistry and Research, University of Vienna, Währinger Straße 42, 1090 Vienna, Austria: andreas.mautner@univie.ac.at*

* To whom correspondence should be addressed.

Michael Hummel: Tel: +358505124198, E-Mail: [michael.hummel@aalto.fi](mailto:michael.hummel@aalto.fi)

Stefan Spirk: Tel: +43 316 87330763, E-Mail: [stefan.spirk@tugraz.at](mailto:stefan.spirk@tugraz.at)

Table S1: Overview of the insignificant model coefficients for each response and corresponding determination coefficients R^2^.

| Response | Insignificant model coefficients | R^2^ |
| --- | --- | --- |
| Mass yield | b_C:KOH_^2^, b_T·C:KOH_ | 0.944 |
| SSA_BET_ | none | 0.942 |
| A_mic_ | none | 0.927 |
| V_meso_ | b_T_^2^, b_C:KOH_^2^, b_T·C:KOH_ | 0.914 |
| C_EA_ | b_C:KOH_^2^, b_T·C:KOH_ | 0.982 |
| N_EA_ | b_C:KOH_, b_T_^2^ , b_C:KOH_^2^, b_T·C:KOH_ | 0.792 |
| O_EA_ | b_C:KOH_, b_T_^2^ , b_C:KOH_^2^, b_T·C:KOH_ | 0.884 |
| C_XPS_ | b_T·C:KOH_ | 0.917 |
| N_XPS_ | all | - |
| O_XPS_ | none | 0.932 |
| I_D_/I_G_ | all | - |

Figure S1: Nitrogen physisorption isotherms at 77 K, color-coded according to activation temperature.

Figure S2: Pore size distribution derived from Dollimore-Heal adsorption data, color-coded according to activation temperature.

Figure S3: (a) Calculated model coefficients for carbon content determined by EA. Main effect plots of (b) activation temperature, (c) C:KOH ratio on the carbon content of the activated carbons and (d) response surface for carbon content determined by EA (%).

Figure S4: (a) Calculated model coefficients for oxygen content determined by EA. Main effect plots of (b) activation temperature, (c) C:KOH ratio on the oxygen content of the activated carbons and (d) response surface for oxygen content determined by EA (%).

Figure S5: SEM images of all activated carbons according to the design matrix with a magnification of 100 X. Samples: (a) 01-AC-700-K3, (b) 02-AC-800-K3, (c) 04-AC-700-K4, (d) 05-AC-800-K4, (e) 06-AC-900-K4, (f) 07-AC-700-K5, (g) 08-AC-800-K5, and (h) 09-AC-900-K5.

Table S2: Averaged I_D_/I_G_ ratios of all samples from three measurements.

| Sample | I_D_/I_G_ |
| --- | --- |
| 01-AC-700-K3 | 0.935 |
| 02-AC-800-K3 | 0.947 |
| 04-AC-700-K4 | 0.923 |
| 05-AC-800-K4 | 0.934 |
| 06-AC-900-K4 | 0.941 |
| 07-AC-700-K5 | 0.934 |
| 08-AC-800-K5 | 0.944 |
| 09-AC-900-K5 | 0.959 |
| 10-AC-800-K4 | 0.962 |
| 11-AC-800-K4 | 0.955 |

Figure S6: Raman spectra normalized on the I_G_ band and color-coded according to activation temperature.
